# Rehabilitation in a Pediatric Patient Who Underwent Correction Surgery for Tetralogy of Fallot: A Case Report

**DOI:** 10.7759/cureus.50442

**Published:** 2023-12-13

**Authors:** Sanjivani S Bangde, Vaishnavi M Thakre, Tejaswini Fating, Aditi Dandekar

**Affiliations:** 1 Community Health Physiotherapy, Ravi Nair Physiotherapy College, Datta Meghe Institute of Higher Education and Research, Wardha, IND

**Keywords:** congenital heart disease, thoracic expansion, physiotherapy, ventricular septal defect, tetralogy of fallot

## Abstract

Tetralogy of Fallot (TOF) is a congenital heart defect characterized by four distinct heart abnormalities, which include an overriding aorta (where the aorta crosses both ventricles), a ventricular septal defect (VSD), right ventricular hypertrophy (the right ventricle muscle is thickened), and pulmonary stenosis (the pulmonary valve and artery are narrowed). Individuals suffering from TOF may exhibit pinkness, cyanosis at baseline, or episodes of hypercyanosis. The pathoanatomy of the TOF allows blood from the pulmonary and systemic circulations to mix. Cyanosis is caused by the addition of deoxygenated blood from a shunt that runs from right to left to the systemic circulation. In this case report, we present a five-year-old female patient with a known case of TOF. The results were recorded using the Pediatric Quality of Life (PedsQL) Questionnaire, New York Heart Association (NYHA) Dyspnoea Scale, Wong-Baker Faces Pain Rating Scale, and arterial blood gas analysis. Therapy goals were to improve overall functional ability, to remove secretions from airway, and the return of acceptable cardiovascular function. This case report focuses on the success of the cardiorespiratory rehabilitation program based on the patient's current state of health. The outcome parameters confirm that patients can experience improved functional recovery.

## Introduction

Congenital heart conditions can be caused by abnormal cardiac structural alterations that start early in pregnancy and persist through delivery. They affect roughly one in 125 babies, making them the most prevalent birth abnormalities [1]. Tetralogy of Fallot (TOF), a congenital heart defect, is characterized by four different heart abnormalities: pulmonary stenosis (narrowing of the pulmonary valve and artery), ventricular septal defect (VSD), right ventricular hypertrophy (thickening of the right ventricle (RV) muscle), and overriding aorta (thickening of the RV wall) [2]. Cyanosis can result from this disorder, which can cause blood to be deficient in oxygen flowing from the heart to the rest of the body. Surgery is frequently used to enhance blood flow and reduce symptoms [3]. TOF reveals aberrant septo-parietal trabeculations and anterior cephalad distortion of the myocardial intraventricular septum, which results in sub-pulmonary infundibular stenosis [4]. Individuals suffering from TOF may exhibit pinkness, cyanosis at baseline, or episodes of hypercyanosis.

The degree of pulmonary stenosis and obstruction of the right ventricular outflow tract (RVOT) determine their physiological functionality. With additional hypertrophy of the RV infundibular muscles, the risk of hypercyanotic episodes increases with time [5]. The pathoanatomy of the TOF allows blood from the pulmonary and systemic circulations to mix [6]. Cyanosis occurs due to a right-to-left shunt that introduces oxygen-deprived blood into the systemic circulation. Near the VSD, this mixing frequently takes place [7]. The right-to-left shunt across the VSD is controlled by the relative pressure gradient between the RV and left ventricle (LV). The right ventricular outflow tract obstruction (RVOTO) degree determines the amount of pulmonary blood flow or the RV stroke volume [8]. Surgical intervention is available as a treatment option for TOF shortly after the infant is born. The pulmonary valve is replaced or the RVOTO is widened during surgery. A closure patch is commonly used to close the opening between both ventricles. The body's overall blood flow, including to the lungs, will be enhanced by these actions [9]. TOF is identified shortly after birth. Infants might have blue skin. While auscultating, a doctor may hear an unusual whooshing sound, often known as a heart murmur [10]. Diagnostic tests for this condition include pulse oximetry, echocardiogram, electrocardiogram, chest X-ray, and cardiac catheterization [11]. To prevent postoperative complications, physiotherapy rehabilitation is commonly utilized. Various physiotherapeutic methods, like postural drainage, positioning, thoracic expansion, and breathing exercises, can help in clearing secretions from the lungs, improve ventilation, and decrease the work of breathing. It also enhances the well-being and the patient's quality of life.

## Case presentation

We present the case of a five-year-old female patient diagnosed with TOF with hypoplastic pulmonary artery stenosis that complained of breathlessness and easy fatigability while doing any activity. Breathlessness was gradual in onset and progressive in nature, which is relieved upon rest and was graded 3 per the New York Heart Association (NYHA) Dyspnoea Scale. She also complains of frequent bluish discoloration of nails and periphery since two months of age. She was admitted in 2021 for an episode of fever, cough, and a cold in a pediatric ward. As her symptoms subsided, the patient was transferred to the cardiac vascular and thoracic surgery (CVTS) ward for the surgical management of TOF. The patient underwent a Blalock-Thomas-Taussig (BT) shunt on October 10, 2021. Since then, she has been on regular follow-up and has come to the hospital for further treatment of her TOF. Investigations like chest X-rays and 2D echo were done. Due to a large subaortic VSD with severe valvular infundibulum and supravalvular pulmonary stenosis, the patient underwent a transannular RVOT patch on October 6, 2023. The patient's primary complaint post surgery was pain at the suture site while breathing, breathlessness, and easy fatigue with limited activities. The pain was gradual in onset, stabbing in nature. Therapy for physical rehabilitation began the day after the procedure.

Clinical examination

**Figure 1:**
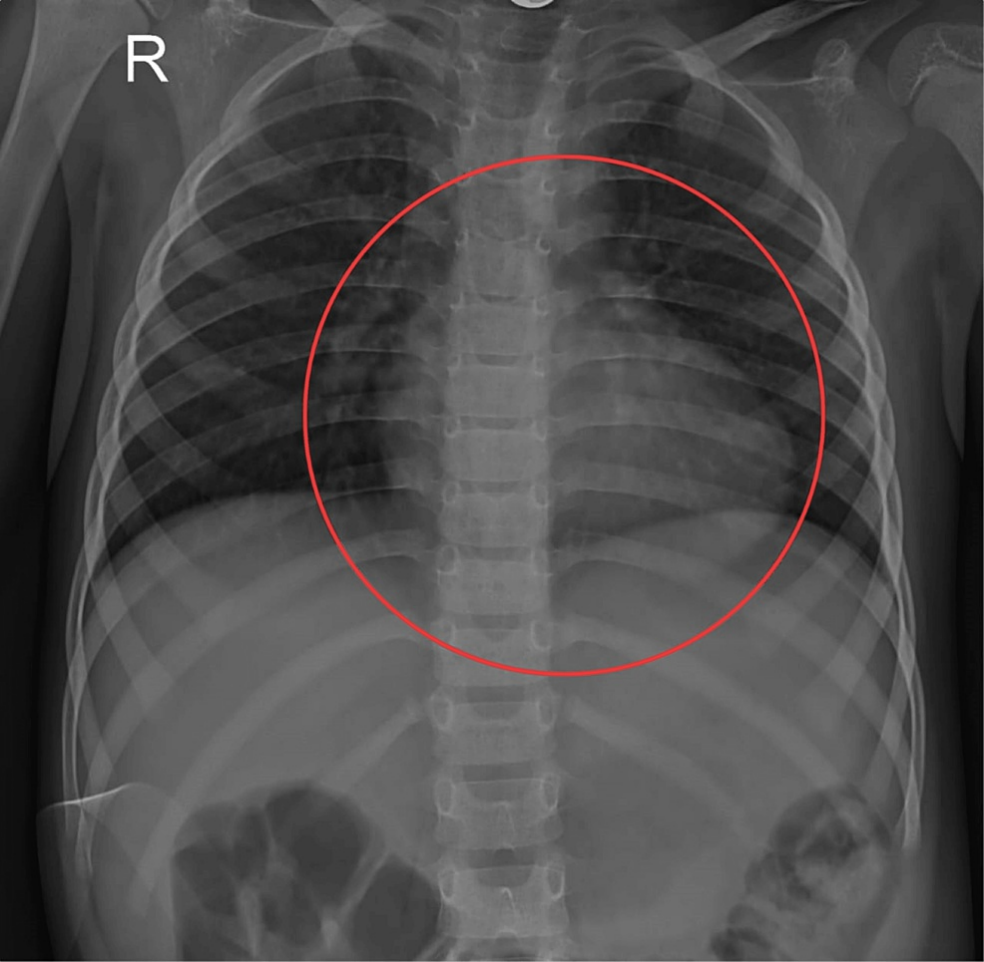
Preoperative chest radiograph: posteroanterior view The circle represents a boot-shaped heart with hyperlucent lungs

**Figure 2:**
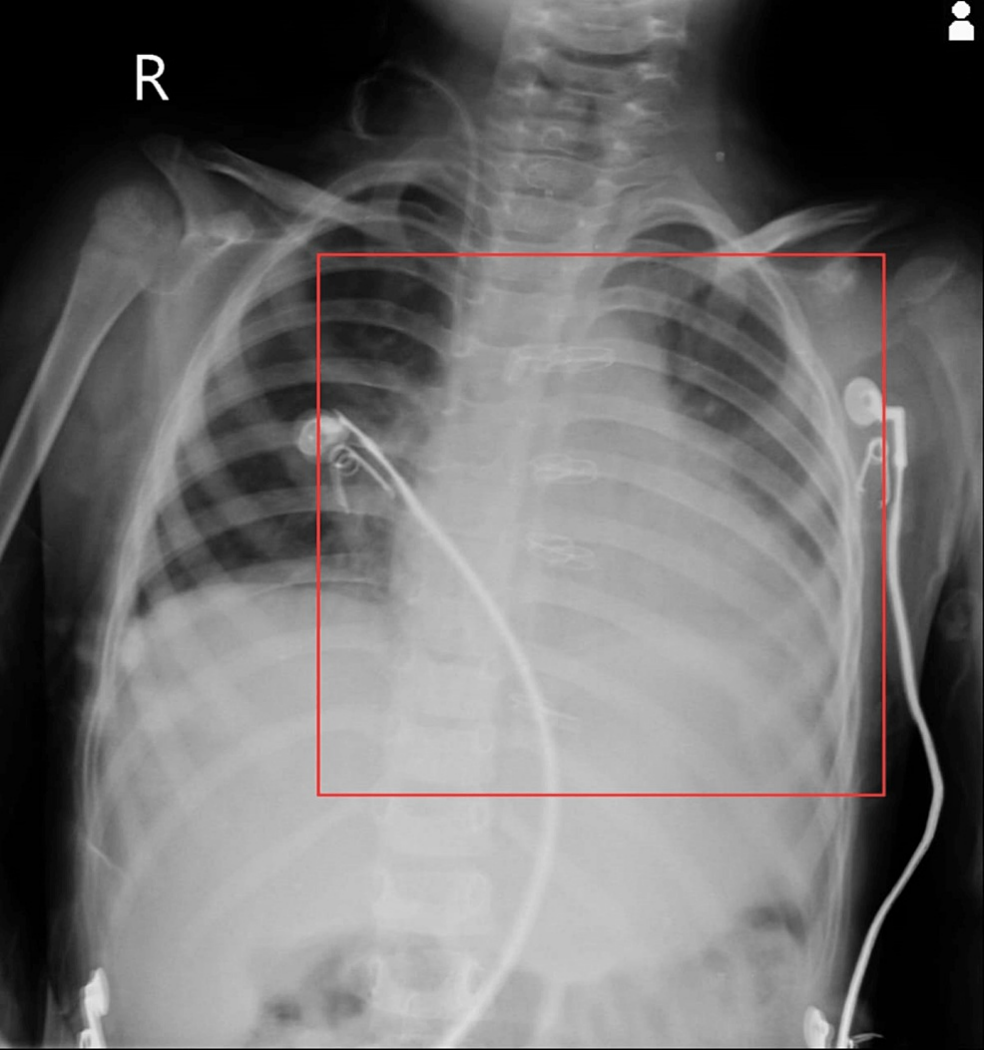
Postoperative chest radiograph Square represents chest leads and cardiomegaly

Consent was obtained from the patient's parents before the beginning of the clinical assessment. On general examination, the patient was conscious, oriented, and cooperative. The patient was afebrile to touch, was hemodynamically stable, and had a mesomorphic build. On inspection, the patient was observed in a sitting position with bilaterally equal chest expansion. The bandage was seen over the sternum from a median sternotomy incision. On palpation, the trachea was centrally placed, and tenderness was present over and around the incision. Dull note was heard on percussion over the upper and lower zones of the lung field. The apex heartbeat was found at the intercostal space, midclavicular line. On auscultation, there were bilateral crepitation and a decrease in air entry in the upper and lower lung. Laboratory evaluations demonstrated that most of the red blood cells in the peripheral smear are normocytic and normochromic and there is only a little anisopoikilocytosis with a few microcyte and pencil cells. Antigen-presenting cells are 142000 cells/mm^3^, according to a cell counter, and there are no hemoparasites visible. Platelets are decreased on smear. The chest X-ray is shown in Figure 1 and Figure 2 for residual VSD, dilated RV, and mild pulmonary stenosis with a peak gradient (PG) of 20 mmHg. A thin pericardial effusion was seen.

Timeline

In October 2023, the patient was admitted to the pediatric ward. The timeline from previous surgery to recent admission and discharge is given in Table 1.

**Table 1:** Timeline of the patient

Timeline	
Previous surgery date	11/10/2021
Admission sate	4/10/2023
Surgery date	6/10/2023
Physical therapy rehabilitation date	8/10/2023
Discharge date	14/10/2023

Therapeutic interventions

Postoperative physiotherapy management focuses on improving breathing patterns, removing secretions, enhancing ventilation, and avoiding physical deconditioning of the patient. Table 2 shows physiotherapy goals and interventions for the five-year-old patient with TOF.

**Table 2:** Postoperative rehabilitation program for patients with therapeutic goals, intervention, and regimen AROM: active range of motion

Physiotherapeutic goals	Physiotherapeutic rehabilitation	Dosage
Patient education	The following physiotherapeutic interventions must be carried out, and caregivers must be educated and counselled regarding the patient's condition	The function of physiotherapists in the patients' treatment was explained to caregivers at the start of the intervention
To enhance ventilation	Positioning the patient in such a way that it reduces the work of breathing and improves ventilation	Every two hours, an alternate position is achieved
To restore lung volume	The patient receives sustained low-frequency rhythmic compression for five seconds at end of inspiration when the physiotherapist places one hand on the anterior chest wall and the other on the posterolateral aspect	Three sets of two to five thoracic compressions each per day are included
To mobilize secretions from terminal bronchioles to the central airway	1) Chest percussion: Based on auscultatory results, a percussor cup is used to tap on different parts of the chest wall	Percussion and vibration are given for two to three minutes bilaterally
	2) Vibrations	
To avoid physical deconditioning	Ambulation	One to two to rounds walk in corridor, two times a day
To improve lung function and capacity and to enhance oxygenation	Pursed lip breathing	10 repetitionx1 set, three times a day
	Paper blowing method	
	Thoracic expansion exercises	
To reduce muscle tension, improve joint stability, and increase flexibility	Bilateral upper limb and lower limb AROM exercises	10 repetitionsx1 set, three times a day

**Figure 3:**
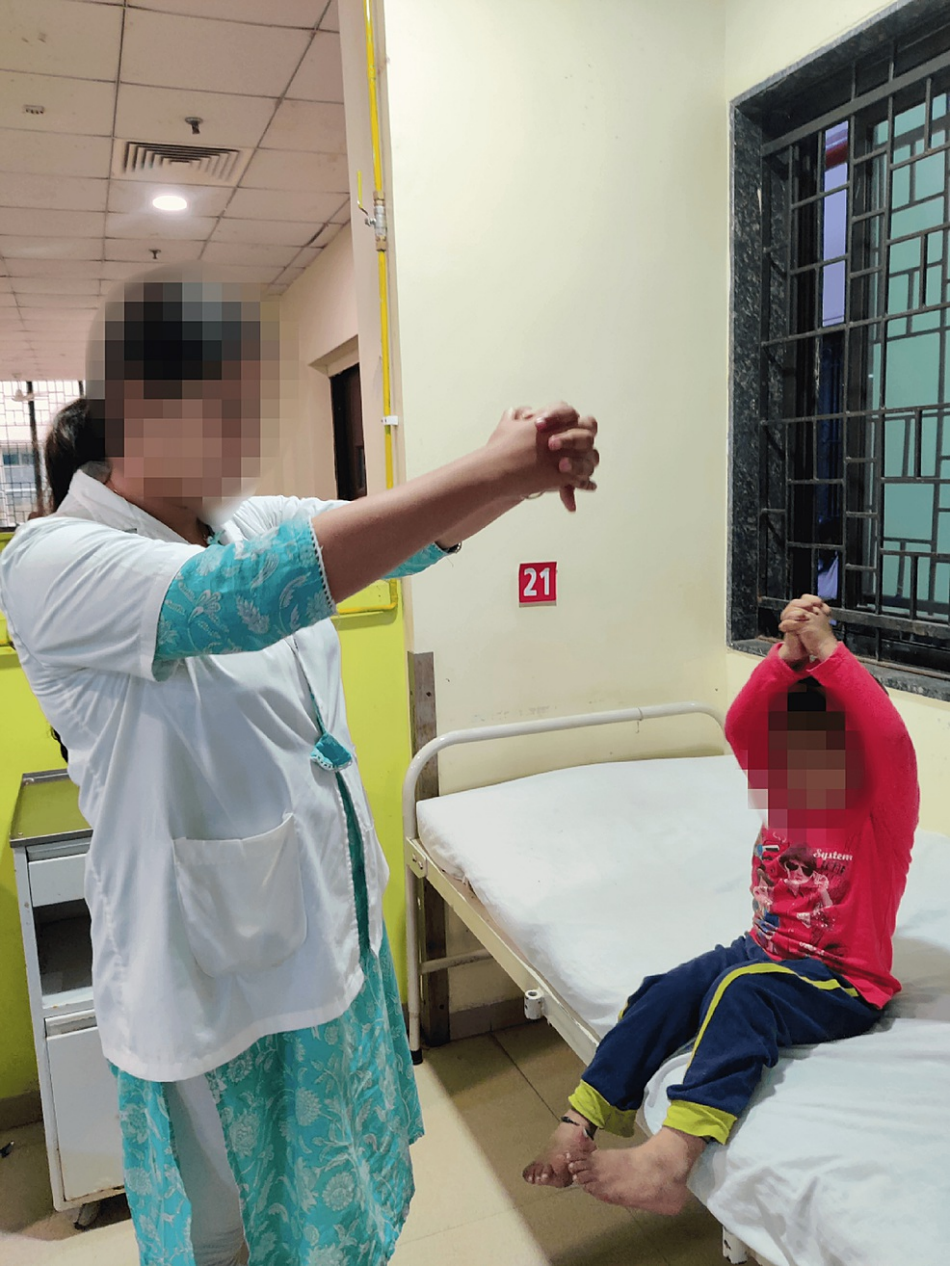
Patient performing thoracic expansion as physiotherapeutic intervention

**Figure 4:**
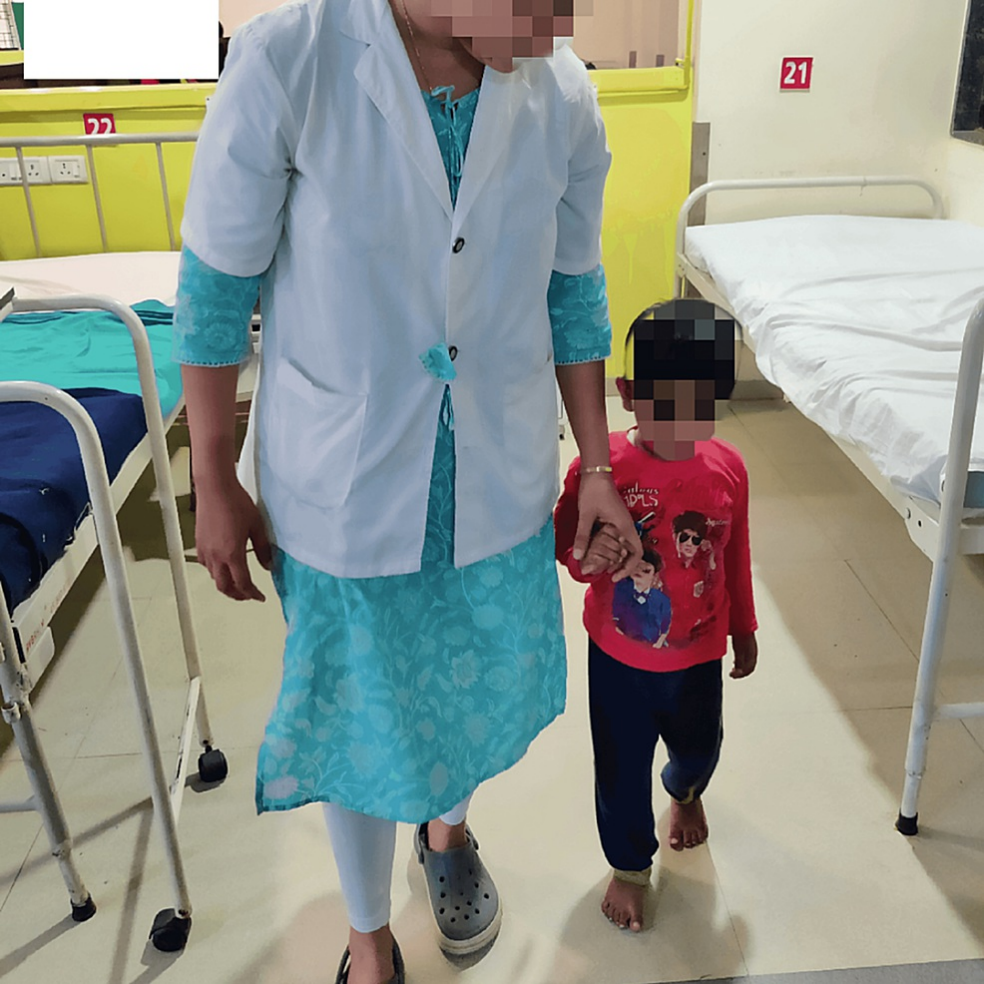
Patient performing ambulation along the hallway

Figure 3 and Figure 4 show the patient performing thoracic expansion exercise to increase the lung capacity and hallway ambulation to prevent deconditioning of the whole body under the therapist's supervision.

Outcome measures

**Figure 5:**
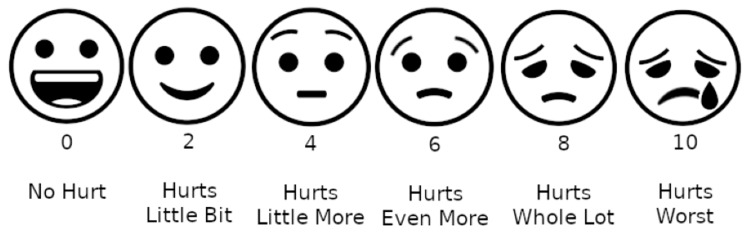
Wong-Baker Faces Pain Rating Scale

The Pediatric Quality of Life (PedsQL) Questionnaire, New York Heart Association (NYHA) Dyspnoea Scale, Wong-Baker Faces Pain Rating Scale (Figure 5; Table 3), and arterial blood gas (ABG) analysis are outcome measures for this case report. Before and after physiotherapy rehabilitation, both parameters were taken. ABG analysis report is shown in Table 4.

**Table 3:** Outcome measures PedsQL: Pediatric Quality of Life; NYHA: New York Heart Association

	Outcome measure scale	Pretreatment score	Posttreatment score
1	PedsQL	49 out of 100	84 out of 100
2	NYHA Dyspnoea Scale	Grade 4	Grade 2
3	Wong-Baker Pain Rating Scale	Hurts even more grade 3	Hurts little bit grade 1

**Table 4:** ABG analysis PaCo2: partial pressure of carbon dioxide; PaO2: partial pressure of oxygen in the arterial blood; HCO3: bicarbonate; SaO2: arterial oxygen saturation; ABG: arterial blood gas

ABG analysis	Postoperative day 1	On the day of discharge
PH	6.589	7.374
PaCo2 (mmHg)	50.2	38.2
PaO2 (mmHg)	114.2	72.6
HCO3 (mmol/L)	27.1	23.8
SaO2 (%)	90	96

## Discussion

According to Rhodes et al., children with congenital heart disease (CHD) usually have reduced exercise capacity. This depression is thought to be caused by persistent hemodynamic defects and physical inactivity-related deconditioning [12]. As suggested by Hock et al., patients with TOF have reduced pulmonary blood flow prior to surgical correction and persistent malfunction of the RV and pulmonary valve throughout their lives, which affects their ability to exercise and their lung volumes. By improving pulmonary blood flow and lung volume through inhalation training, exercise capacity can be increased [13]. Goldberg et al. reported that after surgical repair, physical training can help patients exercise more effectively, allowing them to function at or near-normal levels of activity [14]. Miller et al. found that a supervised physical activity intervention before and after surgery for a young patient scheduled for elective cardiovascular surgery may lead to better results [15]. Following surgery for CHD, exercise training may increase peak VO2 in children and adolescents; therefore, it should be considered for cardiac rehabilitation [16].

Bhatt and colleagues suggested that spirometry testing of pulmonary function may be essential to evaluating TOF. Exercise performance may be enhanced by interventions like exercise rehabilitation programs that enhance chronotropic response and pulmonary health. Interventional studies are required to determine these relationships' causality and assess the effectiveness of potential treatments to increase this population's exercise capacity [17]. Norozi et al. reported that the children who underwent complex heart surgery demonstrated significantly reduced mean normalized maximal performance [18]. However, it was relatively uncommon in those with pulmonary atresia and VSD than in the group with TOF [19]. In this case, treating a patient with various physiotherapeutic interventions like breathing techniques, thoracic expansion, ambulation, active upper limb and lower limb exercise, chest percussion, and vibration leads to improved lung function and increased functional capacity.

## Conclusions

The physiotherapeutic purpose was to improve lung function, remove secretions from the airway, improve the functional capacity of the patient, maintain the airway and breathing, and bring back normal cardiorespiratory function. Cardiorespiratory physiotherapeutic interventions like breathing exercises, thoracic expansion, chest percussions and vibration, and thoracic compressions show positive results in improving the patient's functional capacity. The application of postoperative physiotherapy strategies helps in recovery and avoids complications. Using various outcome measures like PedsQL, ABG analysis report, Wong-Baker Faces Pain Rating Scale, and NYHA Dyspnoea Scale was directed to record the effect of physiotherapeutic treatment and improvement in the patient.
